# Inhibition of PHLPP2/cyclin D1 protein translation contributes to the tumor suppressive effect of NFκB2 (p100)

**DOI:** 10.18632/oncotarget.8746

**Published:** 2016-04-15

**Authors:** Jiawei Xu, Yulei Wang, Xiaohui Hua, Jiheng Xu, Zhongxian Tian, Honglei Jin, Jingxia Li, Xue-Ru Wu, Chuanshu Huang

**Affiliations:** ^1^ Nelson Institute of Environmental Medicine, New York University School of Medicine, Tuxedo, NY 10987, USA; ^2^ Department of Urology, New York University School of Medicine, and Veterans Affairs New York Harbor Healthcare System, Manhattan Campus, New York, NY 10010, USA; ^3^ Current address: Department of Pediatrics, Union Hospital, Tongji Medical College, Huazhong University of Science and Technology, Wuhan 430022, China

**Keywords:** NFκB2 (p100), cyclin D1, PHLPP2, CREB, miR-302d

## Abstract

Although the precursor protein of NFκB2 (p100) is thought to act as a tumor suppressor in mammalian cells, the molecular mechanism of its anti-tumor activity is far from clear. Here, we are, for the first time, to report that p100 protein expression was dramatically decreased in bladder cancers of N-butyl-N-(4-hydroxybutyl)-nitrosamine (BBN)-treated mice and human patients. Knockdown of p100 in cultured human bladder cancer cells promoted anchorage-independent growth accompanied with elevating abundance of cell-cycle-related proteins and accelerated cell-cycle progression. Above effects could be completely reversed by ectopically expression of p100, but not p52. Mechanistically, p100 inhibited Cyclin D1 protein translation by activating the transcription of LARP7 and its hosted miR-302d, which could directly bind to 3′-UTR of cyclin d1 mRNA and inhibited its protein translation. Furthermore, p100 suppressed the expression of PHLPP2 (PH domain and leucine-rich repeat protein phosphatases 2), thus promoting CREB phosphorylation at Ser133 and subsequently leading to miR-302d transcription. Taken together, our studies not only for the first time establish p100 as a key tumor suppressor of bladder cancer growth, but also identify a novel molecular cascade of PHLPP2/CREB/miR-302d that mediates the tumor suppressive function of p100.

## INTRODUCTION

Mammalian nuclear factor κB (NFκB) consists of five members, including RelA/p65, c-Rel, RelB, NFκB1(p50) and NFκB2(p52). NFκB1(p50) and NFκB2(p52) are synthesized as large precursors of p105 and p100, respectively in mammalian cells [1]. While RelA/p50 heterodimer is the predominantly form and plays a major regulatory role in mammalian cells, the RelB/p100 is also expressed and acts as the alterative pathway regulating its downstream gene expression [2]. Although the diverse tumor-promoting roles of NFκB in cancer cell proliferation, anti-apoptosis, angiogenesis, invasion and metastasis, are well established [3–9], much less is known about how p100, a precursor protein of NFκB2, acts as a tumor suppressor in many mammalian cells [10].

Urothelial carcinoma of the bladder (BC) is the fifth most common cancer in industrialized countries and the second most common cancer of the genitourinary tract [11]. Etiology of bladder carcinogenesis involves multiple factors, such as genetic and environmental factors, oncogenes and tumor suppressor genes [12]. Although mutations or chromosomal rearrangements of NFκB2 gene encoding p100 have frequently been observed in many cancer types [10], its expression and role in human BC development has never been explored. Moreover, nothing is known about the molecular mechanisms underlying the p100 regulation of bladder urothelial cell growth. Here we assessed the relative abundance of p100 between human BC tissues and their adjacent normal tissues, and between invasive mouse BC tissues and normal mouse bladder tissues; examined the role of p100 in suppression of anchorage-independent BC cell growth using loss- and gain-expression experimental systems, and explored the molecular mechanisms of p100 suppression of urothelial proliferation and tumorigenesis.

Cyclin D1 is encoded by the CCND1 gene in human and is overexpressed in many cancers [13]. Cyclin D1 overexpression has been shown to correlate with early cancer onset and tumor progression [14, 15]. Cyclin D1 expression and accumulation are induced by growth factors and occur at multiple levels including transcription, translation, and protein stability [16, 17]. NFκB subunits can bind to the cyclin d1 promoter and either stimulate or suppress cyclin d1 gene transcription [18]. However, the role of NFκB2 p100 in Cyclin D1 protein translation has never been reported. Here, we discovered that p100 inhibited Cyclin D1 protein translation *via* suppression of PH domain and leucine-rich repeat protein phosphatases 2 (PHLPP2) expression, thereby leading to activation of CREB/miR-302d axis.

## RESULTS

### p100 expression was downregulated in both mouse and human bladder cancer tissues

**Figure 1:**
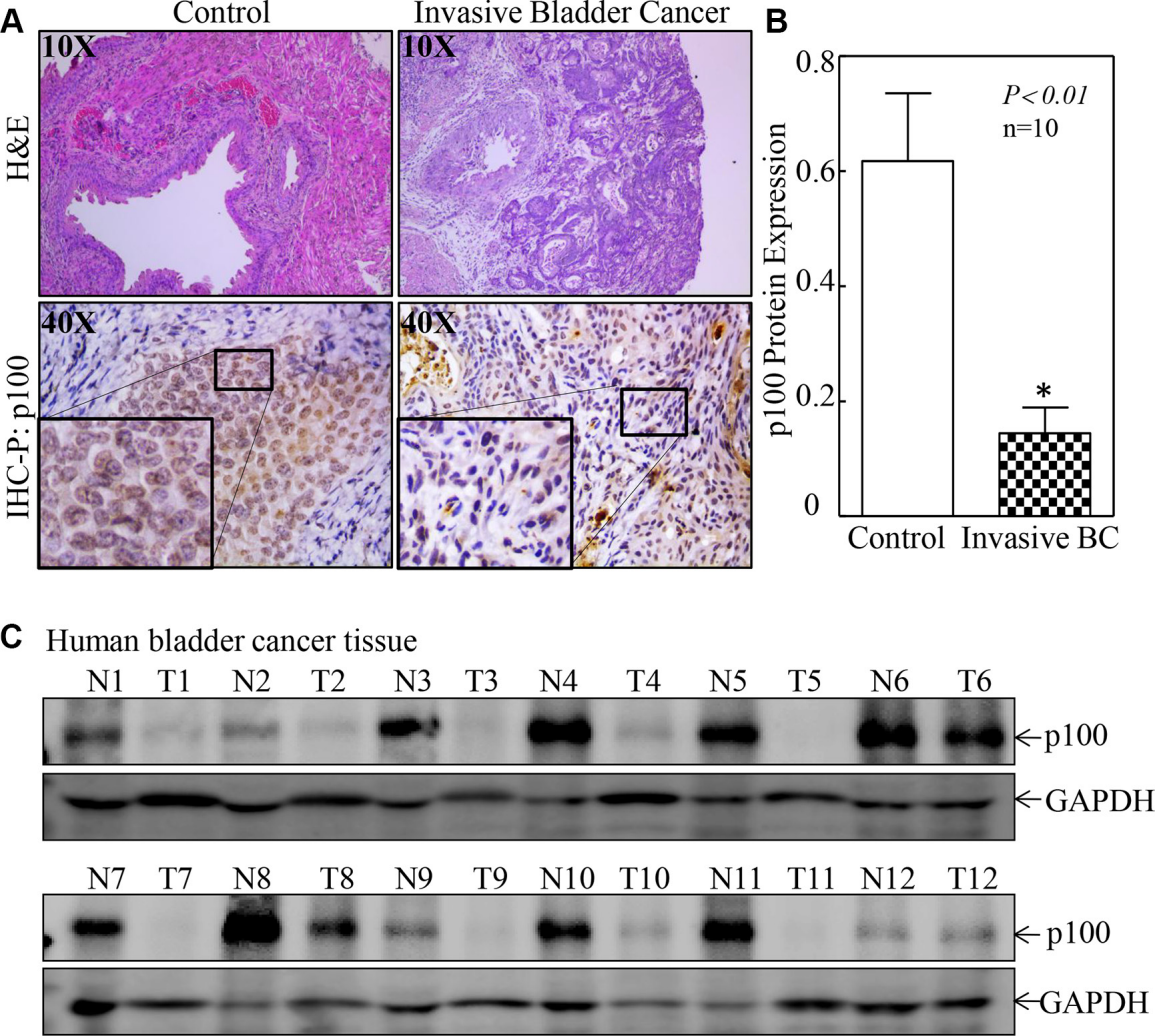
p100 suppression was observed in both mouse and human bladder cancers (**A** and **B**) IHC-P was carried out to evaluate p100 protein expression in mouse bladder cancer tissues as compared with normal bladder tissues. The optical density was analyzed and calculated as described in “Materials and Methods” section (*n* = 10). The symbol (*) indicates a significant difference between two group mice (*p* < 0.01). (**C**) p100 protein expression was evaluated by Western Blot in human bladder cancer tissues and their paired adjacent normal bladder tissues (*n* = 12). GAPDH was used as a protein loading control.

Although NFkB2 is a known tumor suppressor, its expression in bladder cancers has never been explored. To this end, we first examined p100 expression in mouse bladder cancers that was induced by exposure of mice to BBN in drinking water. The results showed that p100 expression was markedly decreased in BBN-induced mouse invasive bladder cancers in comparison to bladder tissues obtained from mice received vehicle-containing drinking (Figure 1A and 1B, *n* = 10, *p* < 0.01). Consistent with this observation in mice, p100 expressions were also decreased in 83.3% of human bladder cancer tissues (10/12) in comparison to their paired adjacent normal bladder tissues (Figure 1C). Our results clearly demonstrate that p100 is downregulated in both mouse and human bladder cancers.

### p100 exhibited an inhibitory effect on anchorage-independent growth and cell cycle progression accompanied with suppression of cyclins in UMUC3 and T24 cells

**Figure 2:**
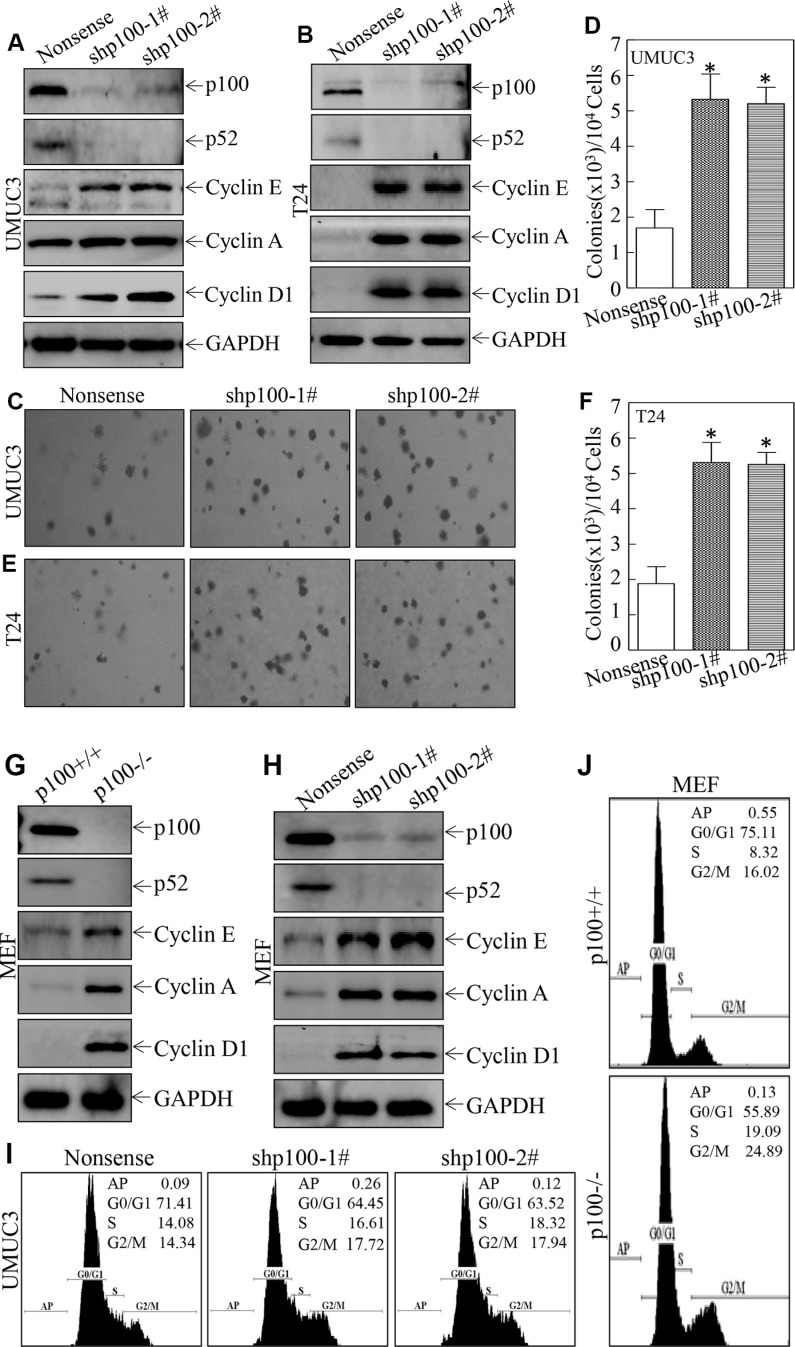
p100 exhibited an inhibitory effect on anchorage-independent growth and cell cycle progression accompanied with suppression of Cyclin in UMUC3 and T24 cells (**A** and **B**) Whole-cell lysates were subjected to Western Blot for determination of protein expression as indicated. GAPDH was used as a loading control. (**C**–**F**) Anchorage-independent growth was determined by soft agar assay as indicated. The number of colonies was scored and presented as colonies per ten thousand cells. The symbol (*) indicates a significant increases in comparison to the control transfectant (*p* < 0.05). (**G** and **H**) Whole-cell lysates were subjected to Western Blot as indicated and GAPDH was used as a loading control. (**J** and **I**) Flow-cytometry analysis of cell cycle alteration was performance as indicated.

We next used shRNA specific targeting human p100 (shp100) to knock down p100 and evaluated the anchorage-independent growth capabilities in UMUC3 and T24 cells. As shown in Figure 2A and 2B, stable transfection of shp100 knocked down expression of both p100 and p52 in UMUC3 and T24 cells, which resulted in a significant promotion of anchorage-independent growth in UMUC3 and T24 cells (Figure 2C–2F), revealing that p100 and/or p52 expression exhibited an inhibition of growth of UMUC3 and T24 cells. To elucidate the mechanisms underlying p100/p52 inhibition of anchorage-independent growth of UMUC3 and T24 cells, we also evaluated the effect of p100/p52 on Cyclin expression. As shown in Figure 2A and 2B, knockdown of p100/p52 expression profoundly led to activation of Cyclin A, Cyclin D1 and Cyclin E, in both UMUC3 and T24 cells. The inhibitory effect of p100/p52 on cyclin expression was convincingly supported by the results obtained from MEFs (Figure 2G and 2H). Consistently, either knockout or knockdown of p100/p52 expression promoted cell cycle progression (Figure 2I and 2J). These results demonstrate that p100 exhibits an inhibitory effect on anchorage-independent growth and cell cycle progression accompanied with suppression of cyclin, further suggesting that p100 downregulation may contribute to human bladder cancer development.

### NFκB2 p100, but not p52, inhibited Cyclin D1 expression, cell cycle progression and anchorage-independent growth in UMUC3 and T24 cells

**Figure 3:**
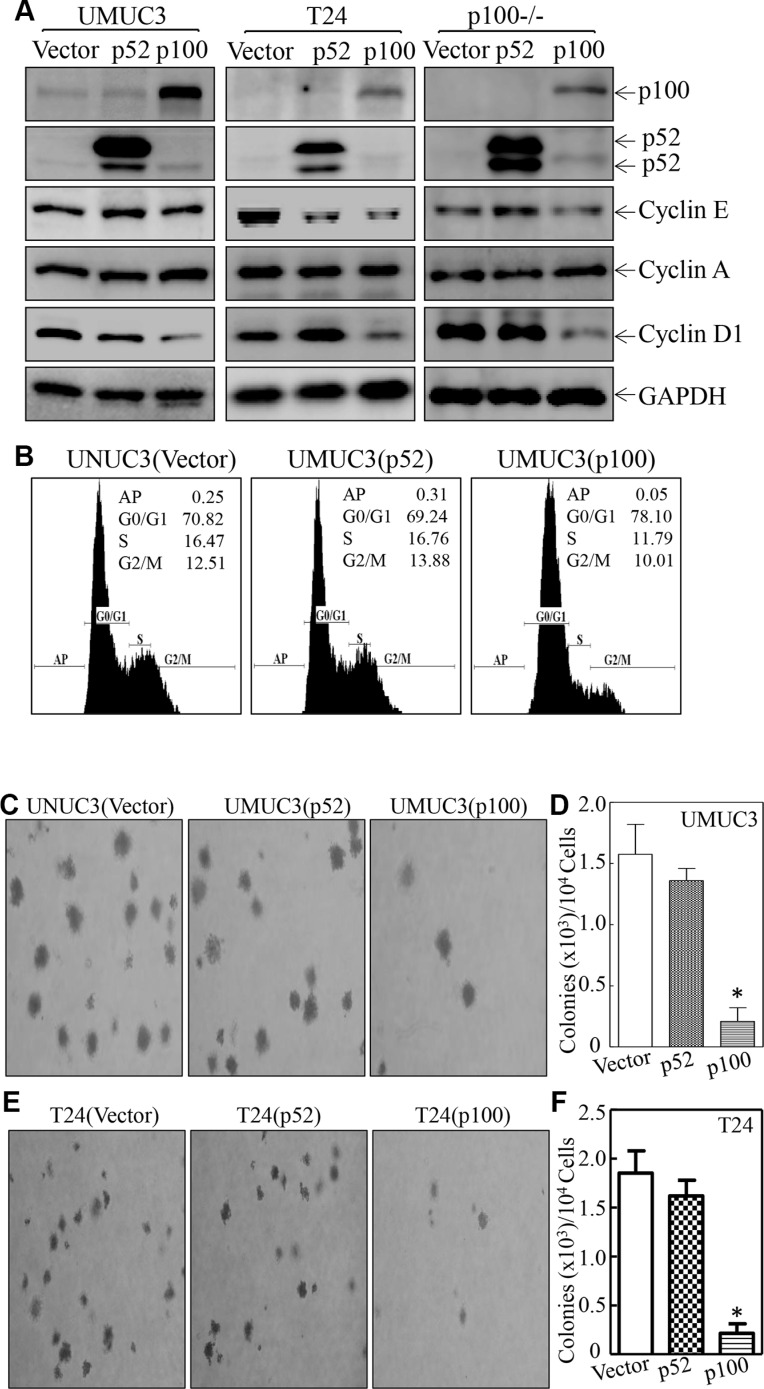

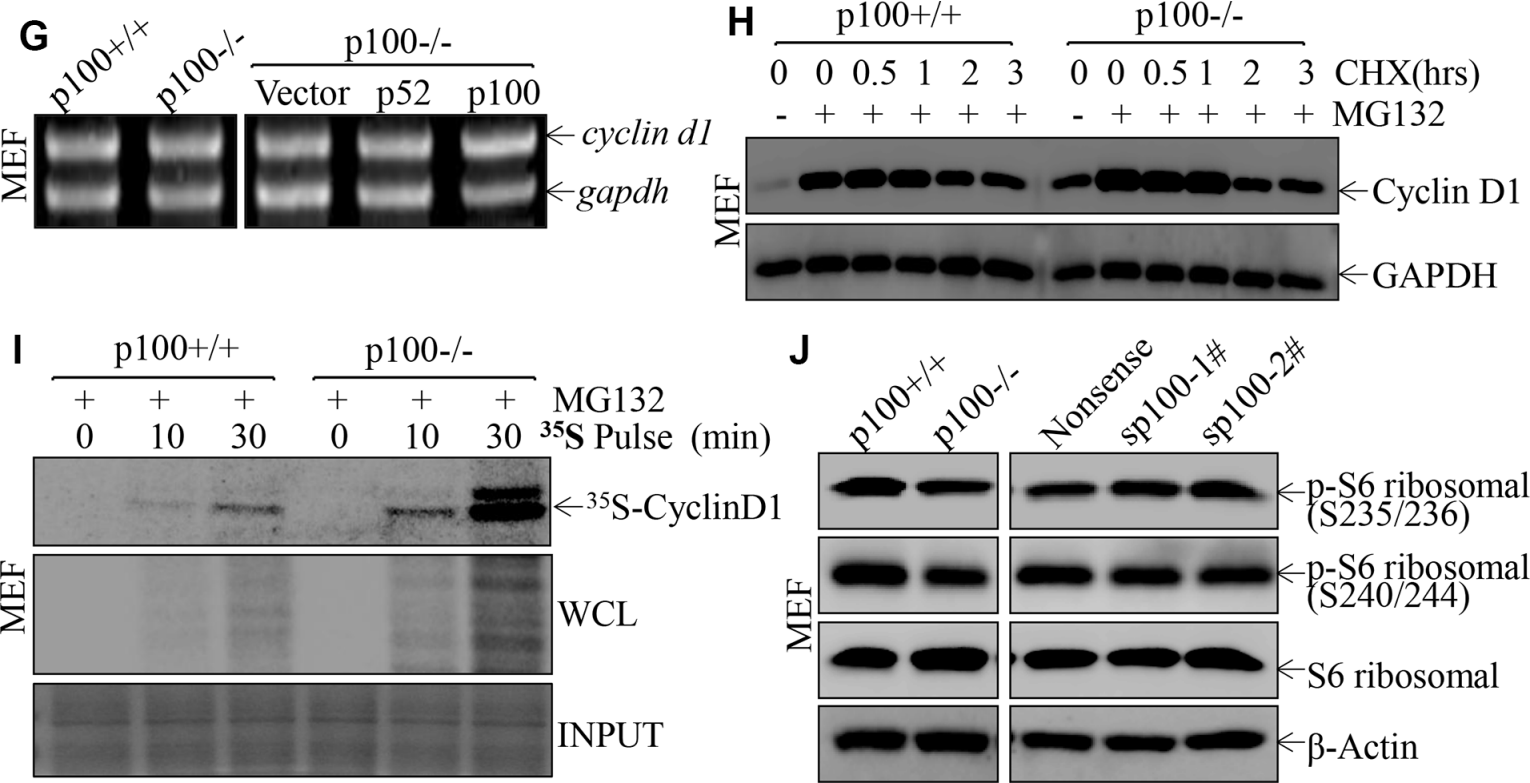
NFκB2 p100, but not p52, mediated the inhibition of Cyclin D1 expression, cell cycle progression and anchorage-independent growth in UMUC3 and T24 cells (**A**) The cell extracts were subjected to Western Blot as indicated. GAPDH was used as a loading control. (**B**) Flow-cytometry analysis of cell cycle alteration was performance as indicated. (**C**–**F**) Anchorage-independent growth was determined in soft agar assay as indicated. The number of colonies was scored and presented as colonies per ten thousand cells. The symbol (*) indicates a significant decreases in comparison to the control transfectant (*p* < 0.05). (**G**) Total RNA was isolated from the indicated cells and then subjected to RT-PCR analysis of cyclin d1 mRNA expression. The gapdh was used as a loading control. (**H**) After pretreatment with MG132 (10 μM) for 4 h, p100+/+ and p100−/− cells were subjected to determination of Cyclin D1 protein degradation in the presence of cycloheximide (CHX) (50 μM). GAPDH was used as a loading control. (**I**) After pre-treatment with MG132 (10 μM) for 30 mins, newly synthesized Cyclin D1 protein in p100+/+ and p100−/− cells was monitored by pulse assay using ^35^S-labeled methionine/cysteine. WCL stands for whole cell lysate. Coomassie blue staining was used for protein loading control. (**J**) The cell extracts were subjected to Western Blot as indicated. β-Actin was used as a loading control.

The p100 has recently been reported to inhibit tumor growth in severe combined immunodeficiency (SCID) mice [10]. Since knockdown of p100 resulted in deficiency of both p100 and p52, our subsequent experiment focused on the identifying which of p100 or p52, was responsible for above inhibition of human bladder cancer cell growth. We have therefore constitutively expressed p100 or p52 in UMUC3 and T24 cells as identified in Figure 3A. Ectopic expression of p100 specific attenuated Cyclin D1 expression in both UMUC3 and T24 cells, while p52 overexpression did not show consistent effect on any Cyclin expression in both cells, suggesting that p100, but not p52, mediated an inhibitory effect on Cyclin D1 expression. This notion was greatly supported by the results obtained from the transfection of p100 or p52 in p100−/− MEFs (Figure 3A). Moreover, overexpression of p100, but not p52, exhibited an inhibition of cell cycle progression in UMUC3 (Figure 3B). In addition, p100 overexpression inhibited anchorage-independent growth of UMUC3 and T24 cells, while p52 did not show such inhibition (Figure 3C–3F). Given that p52 plasmid is a p100 construct with its C-terminal deletion (443–900-aa) [19, 20], above results clearly demonstrate that p100, but not p52, exhibits an inhibition of Cyclin D1 expression, cell cycle progression and anchorage-independent growth in human BC cells.

### p100 inhibited Cyclin D1 protein translation *via* S6 ribosomal-independent pathway

NFκB have been reported to control cellular proliferation by processing tightly coupled to its ability to regulate Cyclin D1 transcription [18]. To elucidate the mechanism of p100 suppression of Cyclin D1 expression, cyclin d1 mRNA level was firstly determined in p100+/+ vs. p100−/− cells, p100−/−(vector) vs. p100−/− (p52) or p100−/− (p100) cells. The results showed that the cyclin d1 mRNA levels were almost comparable among these five cells (Figure 3G), excluding the possibility of p100 suppression of Cyclin D1 at the levels of mRNA transcription or degradation. Followed this, the Cyclin D1 protein degradation rate was compared. As shown in Figure 3H, the Cyclin D1 protein degradation rate was almost similar between p100+/+ and p100−/− cells, excluding the possibility of p100 inhibiting Cyclin D1 protein degradation, indicating that p100 might suppress Cyclin D1 protein translation. To test this, the short term new synthesized protein ^35^S-methionine/cysteine pulse labeling assay was utilized. As shown in Figure 3I, the incorporation of ^35^S-methionine/cysteine into newly synthesized Cyclin D1 protein in p100−/− cells was markedly increased in comparison to p100+/+ cells, demonstrating that p100 inhibited Cyclin D1 protein translation in intact cells.

The phosphorylation of ribosomal protein S6 is critical for its mediating mRNA m7GpppG-Cap-dependent protein translation [21]. Thus, phosphorylation of ribosomal protein S6 at Ser235/236 or Ser240/244 has been evaluated in either p100 knockout or knockdown cells. The results showed that the phosphorylation of ribosomal protein S6 was comparable in all cell lines tested (Figure 3J), revealing that ribosomal S6 was not involved in p100-inhibition of Cyclin D1 protein translation.

### miR-302d was activated by p100 and directly inhibited Cyclin D1 protein translation

**Figure 4:**
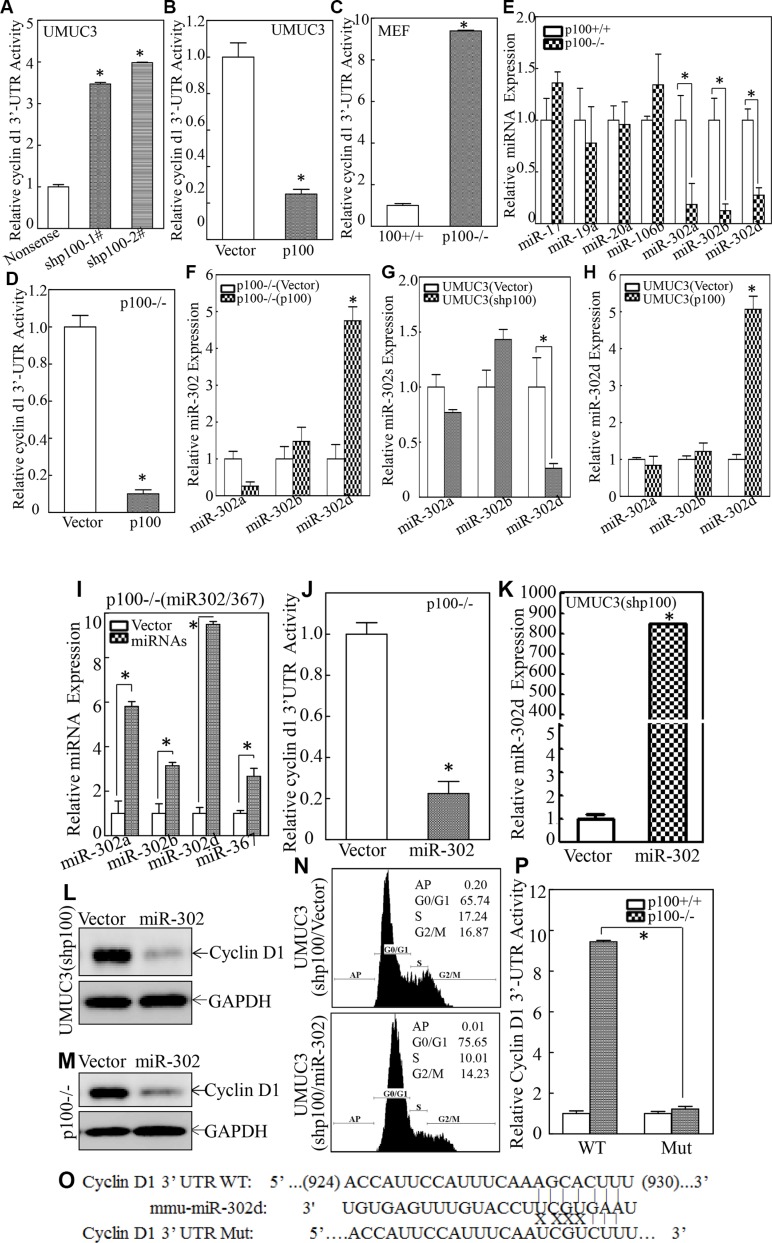
miR-302d was specific activated by p100 and directly inhibited Cyclin D1 protein translation (**A**–**D**) cyclin d1 3′UTR luciferase reporter was transiently transfected into the indicated cells and luciferase activity of each transfectant was evaluated. The results were presented as relative cyclin d1 3′-UTR activity. The symbol (*) indicates a significant difference (*p* < 0.05). (**E**–**H**) The levels of indicated microRNAs were evaluated by quantitative real-time PCR. The symbol (*) indicates a significant difference as compared with control cells as indicated (*p* < 0.05). (**I**) p100−/− cells were stably transfected with construct of miR-302/367 and the miRNA expression levels were determined by real-time PCR. The symbol (*) indicates a significant increase in comparison to the scramble control transfectant (*p* < 0.05). (**J**) The cyclin d1 3′-UTR luciferase reporter was transiently transfected into p100−/−(Vector) or p100(miR-302) cells. Luciferase activity of each transfectant was evaluated and the results were presented as relative cyclin d1 3′-UTR activity. The symbol (*) indicates a significant decrease as compared with that in vector transfectant (*p* < 0.05). (**K**) UMUC3(shp100) cells were stably transfected with construct of miR-302/367. The miR-302d expression was determined by real-time PCR and the symbol (*) indicates a significant increase as compared with control vector transfectant (*p* < 0.05). (**L** and **M**) The cell extracts as indicated were subjected to Western Blot and GAPDH was used as a protein loading control. (**N**) Flow-cytometry analysis of cell cycle alteration was carried out as indicated. (**O**) Schematic of the construction of the cyclin d1 mRNA 3′-UTR luciferase reporter and its mutants were aligned with miR-302d. (**P**) Wild-type and mutant of cyclin d1 3′-UTR luciferase reporters were co-transfected with pRL-TK into p100+/+ and p100−/− cells, respectively. Luciferase activity of each transfectant was evaluated and the results were presented as relative cyclin d1 3′-UTR activity. The symbol (*) indicates a significant decrease in cyclin d1 3′-UTR activity as compared with that in WT cyclin d1 3′-UTR reporter transfectant (*p* < 0.05).

MicroRNAs (miRNAs) are small non-coding RNAs with 20–22 nucleotides [22] and a sequence of 2 to 8 nucleotide long in the miRNA 5′-end region could bind to the 3′-UTR region of its targeting mRNAs, consequently resulting in either mRNA translation suppression or modulation of mRNAs degradation [23]. To test whether miRNA participates in p100 suppression of Cyclin D1 protein translation, cyclin d1 mRNA 3′-UTR-driven luciferase reporter was transiently co-transfected with pRL-TK into either p100 knockdown or ectopic expressed cells, the impact of p100 on cyclin d1 mRNA 3′-UTR-driven luciferase activity was determined. The results indicated that cyclin d1 3′-UTR luciferase activity was significantly increased in UMUC3 (shp100) cells (Figure 4A), while overexpression of p100 dramatically attenuated cyclin d1 3′-UTR luciferase activity in UMUC3 cells (Figure 4B), suggesting that p100 inhibited cyclin d1 3′-UTR luciferase activity. This notion was supported by the results obtained from comparison of cyclin d1 3′-UTR-dependent activity between p100+/+ vs. p100−/− cells or p100−/−(Vector) vs. p100−/−(p100) cells (Figure 4C and 4D). These results revealed that miRNAs might be potential p100 downstream regulators that could bind to cyclin d1 mRNA 3′-UTR and suppress Cyclin D1 protein translation. Then bioinformatics analyses of putative miRNAs binding sites in 3′-UTR of cyclin d1 mRNA were performed, and the results suggested that there were multiple potential miRNAs binding sites in cyclin d1 mRNA 3′-UTR as shown in the Table S2. The expression of these miRNAs were therefore determined in both p100+/+ and p100−/− cells, and the expression of miR-302a, miR-302b and miR-302d was found to be significantly decreased in p100−/− cells, whereas there was no observable difference on miR-17, miR-19a, miR-20a and miR-106b between p100+/+ and p100−/− cells (Figure 4E). The results also showed that ectopic p100 expression in p100−/− cells only restored miR-302d expression (Figure 4F), demonstrating that miR-302d, not miR-302a or miR-302b, was activated by p100. The specific activation of miR-302d by p100 was further extended to UMUC3 cells (Figure 4G and 4H), strongly revealing that p100 is crucial for miR-302d expression.

To determine potential role of miR-302d in the Cyclin D1 expression, a miR-302/367 cluster construct was transfected into p100−/− cells and UMUC3 (shp100) cells, respectively. As shown in Figure 4I, expression of miR-302a, miR-302b, miR-302d and miR-367 was identified in p100−/− cells, and such ectopic expression led to a dramatically inhibition of cyclin d1 3′-UTR activity in p100−/− cells (Figure 4J). Since there is no putative binding site of miR-367 in cyclin d1 3′-UTR luciferase reporter, we anticipated that miR-302d might be a major player in inhibiting cyclin d1 3′-UTR activity. Transfectants of miR-302/367 cluster construct in UMUC3(shp100) cells also expressed a high level of miR-302d (Figure 4K) and inhibited Cyclin D1 expression in both UNUC3 (shp100) and p100−/− cells (Figure 4L and 4M). Constitutive expression of miR302 in UMUC3(shp100) cells induced G_0_/G_1_ growth arrest as compared with UMUC3 (shp100/Vector) cells (Figure 4N). These results demonstrate that miR-302 inhibits cyclin d1 3′-UTR luciferase activity, Cyclin D1 protein expression and cell cycle progression. To provide evidence demonstrating whether miR-302d, rather than miR-302a/miR-302b, direct binds to cyclin d1 mRNA 3′-UTR and inhibits Cyclin D1 protein translation, putative miR-302d binding site in cyclin d1 3′-UTR reporter was point mutated as indicated in Figure 4O. Wild-type (WT) and mutant of cyclin d1 3′-UTR luciferase reporters were transiently co-transfected with pRL-TK into p100+/+ and p100−/− cells, respectively, and the transfectants were used for determination of miR-302d binding site in p100 inhibition of cyclin d1 3′-UTR reporter activity. The results showed that cyclin d1 3′-UTR activity was significantly increased in p100−/− cells as compared with that in p100+/+ cells, whereas the point mutations of miR-302d binding site in cyclin d1 3′-UTR reporter completely abolished the increased luciferase activity in p100−/− cells (Figure 4P). Collectively, our results indicate that miR-302d is able to bind to cyclin d1 mRNA 3′-UTR and inhibit Cyclin D1 protein translation, and cell cycle progression in human BC cells.

### miR-302d was crucial for p100-inhibition of Cyclin D1 protein translation, cell cycle progression and anchorage-independent growth in UMUC3 cells

**Figure 5:**
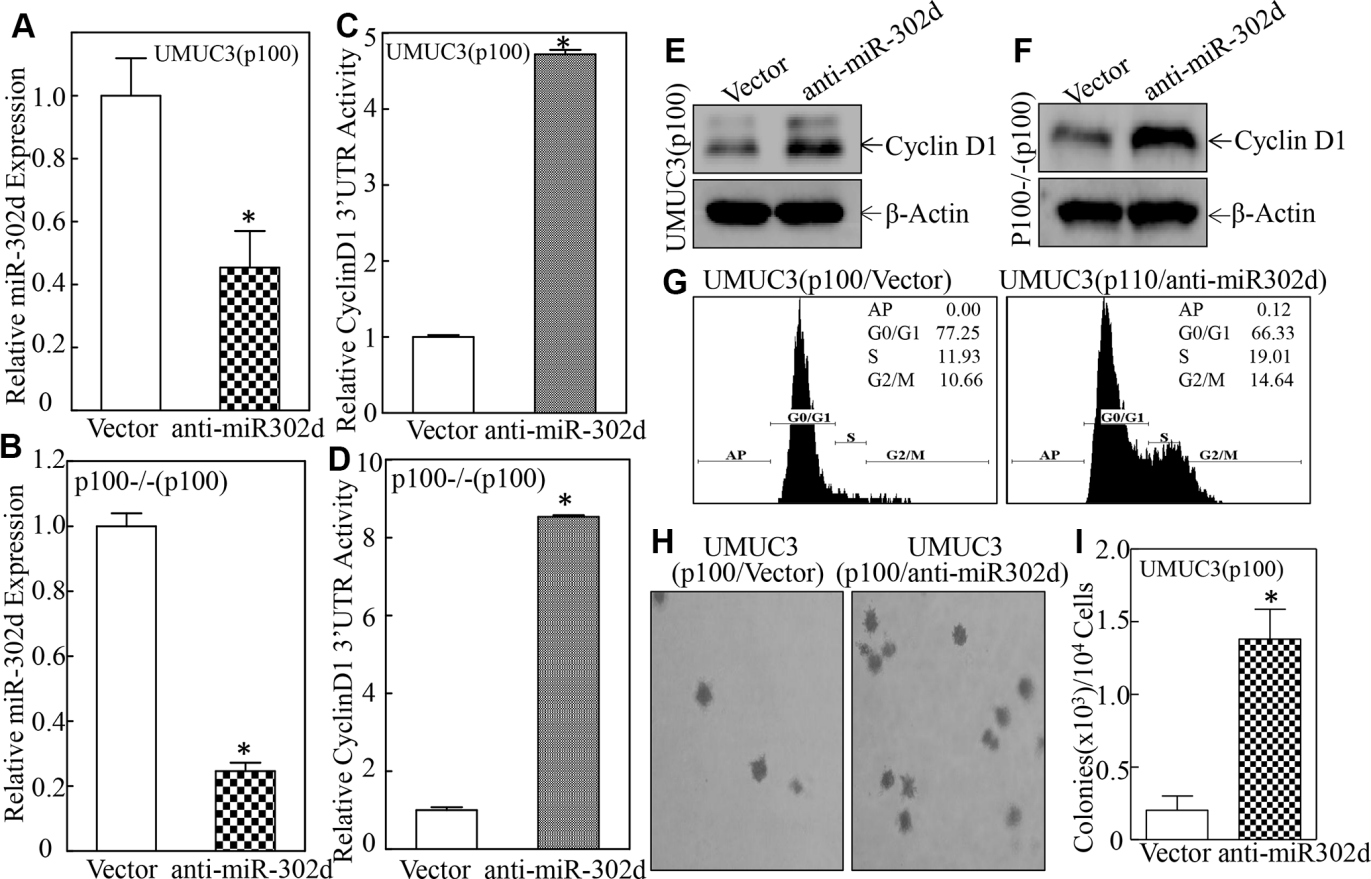
Inhibition of miR-302d reversed p100 attenuation of Cyclin D1 protein expression, anchorage-independent growth, and promoted G0/G1 cell-cycle progression (**A**–**D**) UMUC3(p100) cells and p100−/− (p100) cells were stably transfected with construct of anti-miR-302d and the miR-302d were determined by quantitative real-time PCR. The symbol (*) indicates a significant inhibition of miR-302d expression as compared with vector transfectant (*p* < 0.05) (A and B). The cyclin d1 3′UTR luciferase reporter was transiently transfected into indicated cells and luciferase activity of each transfectant was evaluated. The results were presented as relative cyclin D1 3′-UTR activity and the symbol (*) indicates a significant increase in cyclin D1 3′-UTR luciferase activity as compared with that in vector transfectant (*p* < 0.05) (C and D). (**E** and **F**) The cell extracts were subjected to Western Blot and β-Actin was used as a protein loading control. (**G**) Flow-cytometry analysis of cell cycle was performed as indicated. (**H** and **I**) Anchorage-independent growth was determined in soft agar. And the number of colonies was scored and presented as colonies per ten thousand cells. The symbol (*) indicates a significant increases in comparison to the vector transfectant (*p* < 0.05).

To elucidate the role of miR-302d in p100 inhibition of Cyclin D1 protein translation, miR-302d inhibitor was used to silence miR-302d in UMUC3(p100) and p100−/−(p100) cells. As shown in Figure 5A and 5B, the transfection of antagonizing miR-302d in UMUC3(p100) or p100−/−(p100) cells attenuated miR-302d expression as compared with its corresponding vector transfectants. The antagonizing miR-302d remarkably increased cyclin d1 3′-UTR activity in UMUC3(p100)cells and p100−/−(p100) cells (Figure 5C and 5D), consequently restored Cyclin D1 protein expression (Figure 5E and 5F), in turn promoting cell cycle progression (Figure 5G) and anchorage-independent growth of UMUC3 cells (Figure 5H and 5I). These results clearly reveal that p100 activated miR-302d expression is able to inhibit Cyclin D1 protein translation, cell cycle progression and anchorage-independent growth in human BC cells.

### p100 initiated the transcription of miR-302d and its host gene LARP7 *via* increasing CREB phosphorylation at Ser133

**Figure 6:**
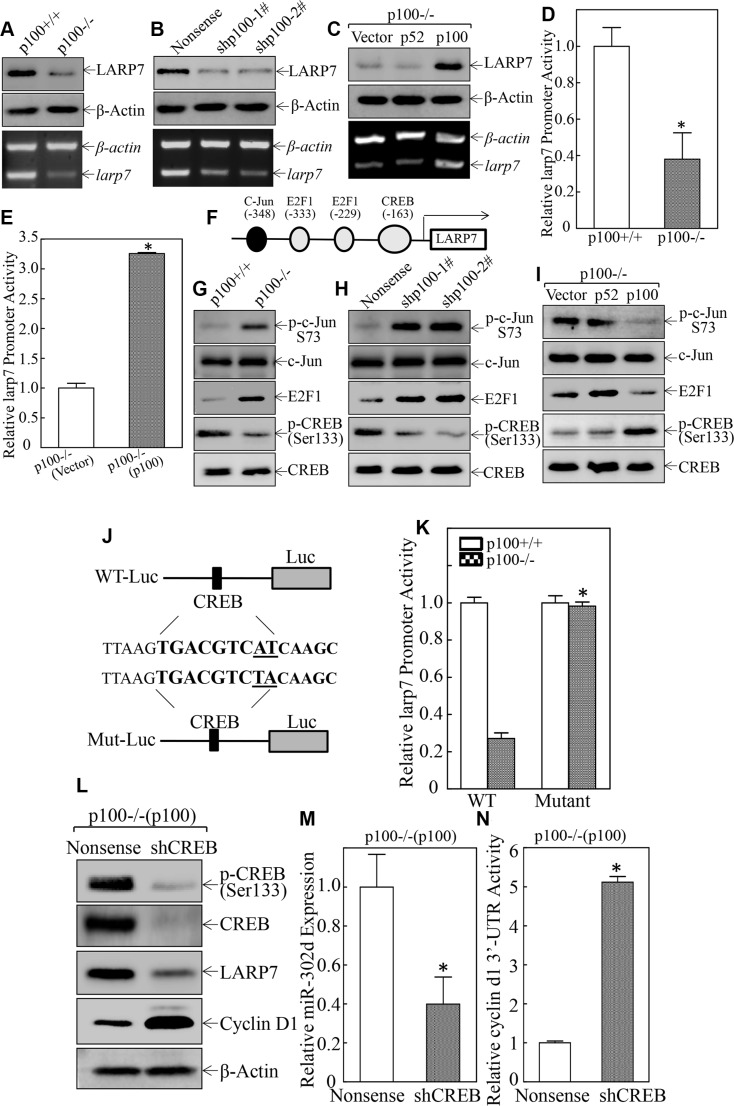
p100 stimulated miR-302d and its host gene LARP7 transcription via inducing CREB phosphorylation at Ser133 (**A**–**C**) The cell extracts as indicated were subjected to Western Blot and β-Actin was used as a protein loading control (top panel). Total RNA was isolated and was then subjected to RT-PCR analysis of larp7 mRNA expression in the indicated cells and β-actin mRNA was used as a loading control (bottom panel). (**D** and **E**) The larp7 promoter-driven luciferase reporter was transiently transfected into the cells as indicated and luciferase activity of each transfectant was evaluated upon seeded in 96-well plate for 48 hrs. The results were presented as relative larp7 promoter activity and the symbol (*) indicates a significant difference of larp7 promoter activities between two groups (*p* < 0.05). (**F**) Schematic representation of the transcription factors binding sites in the mouse larp7 promoter-driven luciferase reporter. (**G**–**I**) The cell extracts as indicated were subjected to Western Blot and β-Actin was used as a protein loading control. (**J**) Schematic representation of CREB point mutant of the larp7/miR302d promoter-driven luciferase reporter. (**K**) Wild-type larp7/miR-302d promoter-driven luciferase reporter or its mutant at CREB binding site, were co-transfected with pRL-TK into p100+/+ and p100−/− cells. Luciferase activity of each transfectant was evaluated and the results were presented as relative larp7 promoter activity. The symbol (*) indicates a significant difference (*p* < 0.05). (**L**) The cell extracts as indicated were subjected to Western Blot and β-Actin was used as a protein loading control. (**M**) The miR-302d expression in the indicated cells was determined by real-time PCR and the symbol (*) indicates a significant inhibition of miR-302d expression as compared with vector transfectant (*p* < 0.05). (**N**) cyclin d1 3′-UTR luciferase reporter was transiently transfected into p100−/−(p100/Nonsense) and p100−/−(p100/shCREB) cells. Luciferase activity of each transfectant was evaluated and the results were presented as relative cyclin d1 3′-UTR activity. The symbol (*) indicates a significant difference of cyclin d1 3′-UTR activities in comparison to p100−/−(Nonsense) cells (*p* < 0.05).

Primary transcript miRNA (pri-miRNA) is generated by RNA polymerase II and cleaved by a nuclear complex formed by Drosha and DGCR8 [24]. Some pri-miRNAs produce miRNAs only (intergenic miRNAs), while others encode miRNAs in the intronic regions of protein-coding host genes (intragenic miRNAs) [25]. The intronic miRNAs and host gene mRNAs are likely co-transcribed and expressed [26]. Analysis of sequence alignment of miR-302d promoter region reveals that miR-302d is an intrinsic miRNA that is localized on chromosome 4 in humans and on chromosome 3 in mice, both within intron 8 of the La ribonucleoprotein domain family member 7 (LARP7) genes [27]. LARP7 belongs to the LARP RNA-binding protein family, and modulates the metabolism and function of a variety of RNA species [28]. We found that LARP7 protein expression was dramatically decreased in either p100 knockout or knockdown cells (Figure 6A and 6B), whereas re-constitutive expression of p100 restored LARP7 protein expression in p100−/− cells (Figure 6C), suggesting that p100 is a positive regulator for LARP7 expression. LARP7 mRNA also showed a consistent alterations with protein expression in all p100 knockout, knockdown, and constitutive transfectants (Figure 6A–6C, bottom panel), indicating that p100 may activate LARP7/miR-302d transcription. This notion was supported by the results showing that p100 deletion led to remarkably inhibition of LARP7 promoter activity, while ectopic expression of p100 in p100−/− cells completely restored LARP7 promoter activity (Figure 6D and 6E).

To evaluate the mechanisms underlying p100 activation of LARP7/miR-302d promoter transcription, the TFANSFAC^®^ Transcription Factor Binding Sites Software (Version 7.0) was used. The results revealed that promoter region of the mouse LARP7 gene contained multiple putative DNA-binding sites for the transcription factors, including c-Jun, E2F1 and cAMP response element-binding protein (CREB) (Figure 6F). To identify the specific transcription factors that participate in p100 activation of LARP7/miR-302d transcription, the transcription factors that have potential binding sites in LARP7/miR-302d promoter were evaluated. The results indicated that c-Jun phosphorylation at Ser73 and E2F1 expression were increased in p100−/− and p100+/+(shp100) cells, and decreased in p100−/−(p100) cells, as compared with their scramble transfectants (Figure 6G–6I). Moreover, total protein expression of CREB and c-Jun were comparable in all transfectants (Figure 6G–6I). In contrast, CREB phosphorylation at Ser133 was inhibited in p100−/− and p100+/+(shp100) cells (Figure 6G and 6H), while ectopic expression of p100, but not p52, rescued CREB phosphorylation at Ser133 in p100−/− cells (Figure 6I). These results reveal that p100 increased CREB phosphorylation at Ser133 was consistent with p100 activation of LARP7/miR-302d, anticipating that CREB might be the transcription factor for p100 activation of LARP7/miR-302d. To test this, the proximal CREB binding site in the LARP7/miR-302d promoter was point mutated as indicated in Figure 6J. Both the mutant and WT of larp7/miR-302d promoter-driven luciferase reporters were transfected into p100+/+ and p100−/− cells, respectively, and the transfectants were used to evaluate role of CREB binding site in p100 regulation of this promoter activity. The results showed that larp7 promoter activity was significantly attenuated in p100−/− cells as compared with that in the p100+/+ cells, while this inhibition in p100−/− cells was not observed in p100−/− cells transfected with CREB binding site point-mutated larp7 promoter-driven luciferase reporter (Figure 6K), suggesting that CREB binding site in LARP7/miR-302d promoter was crucial for p100 activation of LARP7/miR-302d transcription. The p100-stimulation of CREB phosphorylation in its activation of LARP7/miR-302 transcription was convincingly demonstrated by using CREB knockdown in p100−/−(p100) cells. The results indicated that specific knockdown of CREB in p100−/−(p100) cells, resulted in an attenuation of LARP7 protein and miR-302d expression with stimulation of cyclin d1 mRNA 3′-UTR activity and Cyclin D1 protein expression in comparison to its scramble transfectant, p100−/−(p100/Nonsense) (Figure 6L–6N). Collectively, our results demonstrate that p100 increases CREB phosphorylation at Ser133, which consequently binds to promoter region of LARP7/miR-302d and initiates the transcription and expression of miR-302d, and in turn targeting cyclin d1 mRNA 3′-UTR and inhibiting Cyclin D1 protein translation.

### PHLPP2 was a p100 downstream phosphatase being responsible for its mediating CREB phosphorylation at Ser133, and in turn regulated miR-302d/Cyclin D1 axis

**Figure 7:**
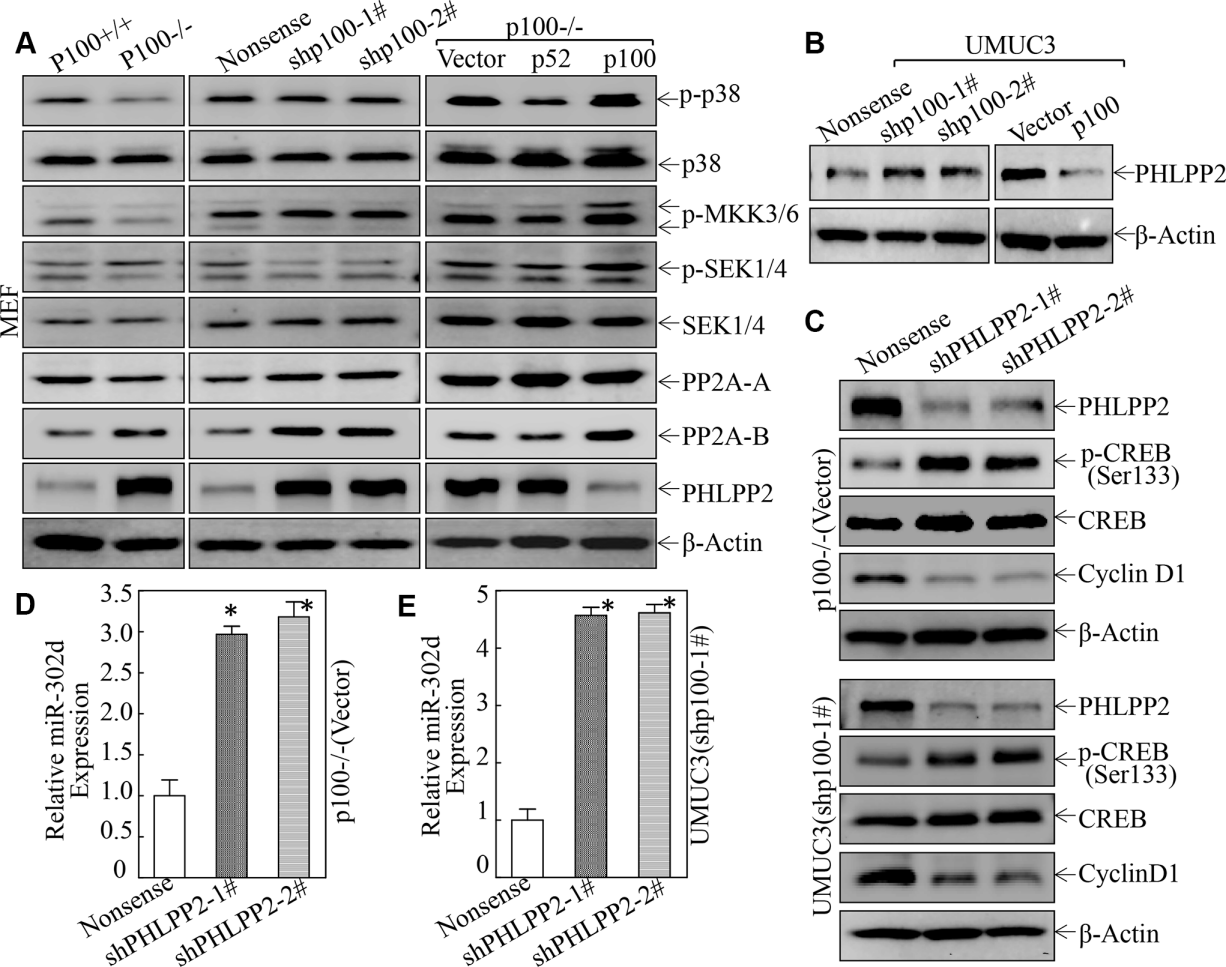
PHLPP2 was a p100 downstream Phosphatase being responsible for its mediating CREB phosphorylation at Ser133, and in turn regulated miR-302d/Cyclin D1 Axis (**A**–**C**) The cell extracts were subjected to Western Blot and β-Actin was used as a protein loading control. (**D** and **E**) The miR-302d expression in the indicated cells was determined by real-time PCR and the symbol (*) indicates a significant increased miR-302d expression as compared with vector transfectant (*p* < 0.05).

Protein phosphorylation could be mediated by either its kinases or phosphatases. The MAP kinases, such as p38, have been reported to act as upstream kinases mediating CREB phosphorylation at Ser133 [29, 30]. We, therefore, investigated the effect of p100 on MAPK and their upstream kinase activation. The results showed that phosphorylation of p38 and its related upstream kinases, such as MKK3/6, SEK1/4, did not show consistent alterations in various cellular systems tested, indicating that these kinases might not be involved in p100 activation of CREB phosphorylation at Ser133 (Figure 7A). Although PP2A (Protein phosphatase type 2A-A) and PP2A-B have been reported to dephosphorylate CREB phosphorylation at Ser133 [31], the results from our studies also excluded their involvement in p100 activation of CREB phosphorylation at Ser133 (Figure 7A). The PHLPP2 is an important regulator of Akt serine-threonine kinases and protein kinase C (PKC) [32]. Since Akt has been shown to modulate CREB phosphorylation at Ser133 [33], we tested whether PHLPP2 was involved in p100-mediated activation of CREB phosphorylation at Ser133. The results showed that PHLPP2 expression was remarkably increased in either p100 knockout or p100 knockdown cells, whereas the restoration of p100 expression in p100−/− cells abolished PHLPP2 expression (Figure 7A). Consistently, PHLPP2 expression was also increased in UMUC3(shp100) cells, while it was dramatically inhibited in UMUC3(p100) cells (Figure 7B). To evaluate role in p100-suppression of PHLPP2 in CREB phosphorylation at Ser133, PHLPP2 was knocked down in p100−/− cells and UMUC3(shp100) cells. As shown in Figure 7C, knockdown of PHLPP2 showed remarkable activation of CREB phosphorylation at Ser133 and miR-302d expression with profound inhibition of Cyclin D1 protein expression in both p100−/− and UMUC3(shp100) cells (Figure 7C–7E). These results demonstrate that PHLPP2 is a p100 downstream phosphatase being mediation of CREB phosphorylation at Ser133, by which activates miR-302d transcription, and in turn targeting cyclin d1 mRNA 3′-UTR and inhibiting Cyclin D1 protein translation.

## DISCUSSION

**Figure 8:**
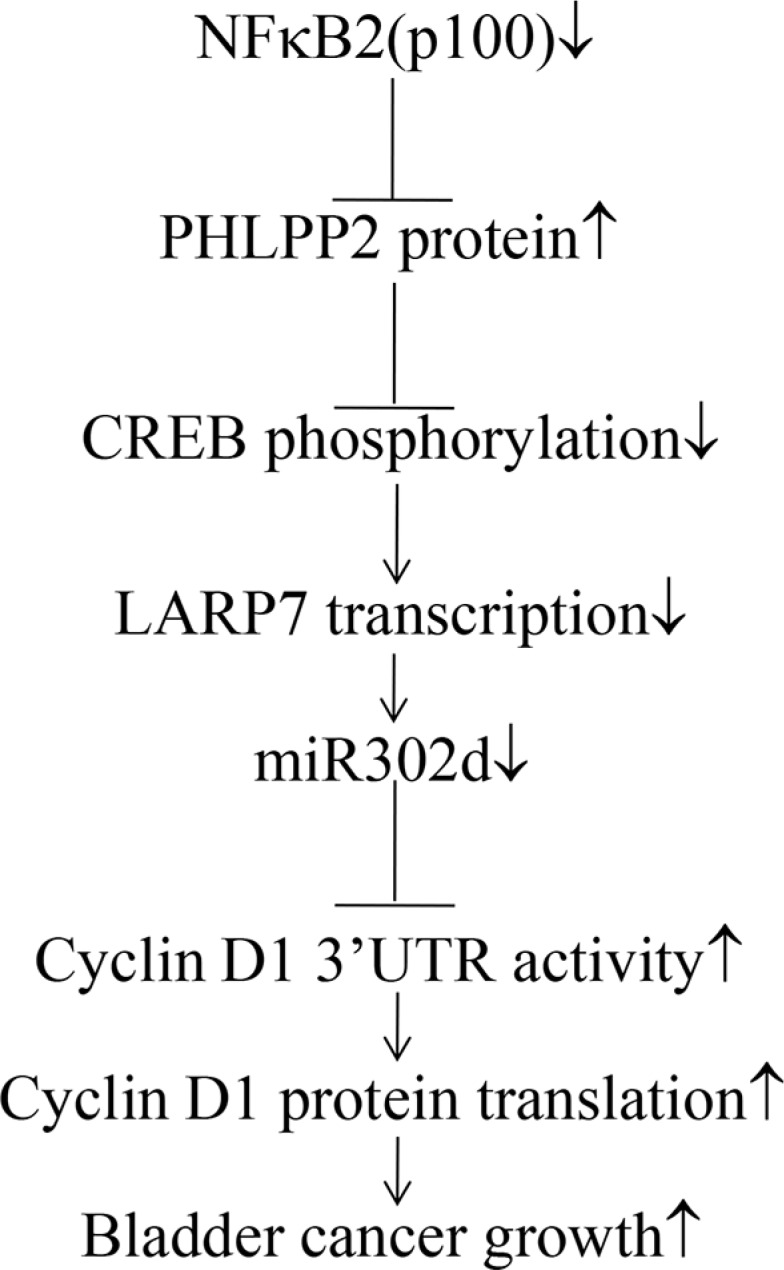
The molecular mechanisms underlying the effect of p100 on bladder cancer development

Although tumor suppressive function of p100 is thought to be associated with its inhibitory role in NFκB activation [34, 35], its expression and association with bladder cancer have never been explored in any previous studies. Our current study discovered that p100 protein expressions were decreased in over 83.3% of human bladder cancers and in all BBN-induced invasive mouse bladder cancers. Knockdown of p100 significantly enhanced anchorage-independent growth of human bladder cancer cells, also accompanied with activation of Cyclin D1 expression and promotion of the cell cycle progression in UMUC3 and T24 cells. The results obtained from gain-expression indicated that constitutive expression of p100, but not p52, exhibited an inhibition of anchorage-independent growth with reduction of Cyclin D1 expression and induction of G_0_/G_1_ growth arrest, indicating that p100 mediated above regulatory effect. Then we revealed that p100 specific inhibited Cyclin D1 protein translation by activation of miR-302d transcription, which was mediated by increased CREB phosphorylation at Ser133. PHLPP2 inhibition was responsible for p100 stimulation of CREB phosphorylation at Ser133. Our results discover p100 protein suppression in human bladder cancers and BBN-induced mouse bladder cancers, also demonstrate that p100 exhibits its tumor suppressive effect on bladder cancer, through a novel mechanism of targeting PHLPP2/CREB/miR-302d axis of inhibition on Cyclin D1 protein translation as diagramed in Figure 8.

Human Cyclin D1 is a protein encoded by the CCND1 gene and is an important regulator of cell cycle progression [14]. Cyclin D1 overexpression has been correlated with early cancer onset and tumor progression in many cancers [14, 36]. Cyclin D1 expression and accumulation are induced by growth factors and occur at multiple levels including increased transcription, translation, and protein stability [17]. Since NFκB can bind to the cyclin d1 promoter region, early studies mainly focus on NFκB regulation of cyclin d1 transcription [18, 37, 38]. Recent studies also show that Cyclin D1 overexpression in cancers does not occur solely as a consequence of gene transcription and amplification, also results from regulation at the post-translational level in several cancers [39]. The Skp2 F-box protein is reported to be involved in modulation of Cyclin D1 degradation [40]. However, nothing is known, to the best of our knowledge, whether p100 can act as a tumor suppressor by attenuating Cyclin D1 protein translation. Here we reported a novel function of p100 in suppressing Cyclin D1 protein translation. We showed that the Cyclin D1 protein was increased in p100−/− MEFs or p100-knockdown in UMUC3 and T24 cells. Cyclin D1 protein upregulation in the p100−/− cells occurred at protein translation level and could be completely abolished by ectopic expression of p100. Furthermore, miR-302d was identified to act as p100 downstream regulator directly being responsible for p100 suppression of Cyclin D1 protein translation.

miRNA plays a key role in the suppression of protein translation by binding to 3′-UTR of its targeted mRNA. The potential miRNA of miR-17/miR-20a cluster has been reported to inversely correlate to Cyclin D1 abundance in human breast cell lines [41]. However, our results indicated that p100 deletion failed to affect expression of miR-17/miR-20 although p100 exhibited an inhibitory effect on cyclin d1 mRNA 3′-UTR activity. In contrast to no effect of p100 on expression of miR-17/miR-20, the results from using p100 knockdown and constitutive expression revealed that p100 was crucial for miR-302d expression. From investigation in effect of miRNA overexpression and point mutation of miR-302d binding site in cyclin d1 mRNA 3′-UTR reporter, we identified that p100-mediated miR-302d was able to directly bind to cyclin d1 mRNA 3′-UTR and inhibit Cyclin D1 protein translation. Our finding is supported by previous report that miR-302 expression suppresses Cyclin D1-CDK4/6 pathways [42].

Many mammalian miRNAs are located within introns of protein-coding genes and they are typically coordinately expressed and processed with the precursor mRNA where they reside [25]. Thus, many intronic miRNAs and host gene mRNAs are likely co-expressed. The mature form of miR-302d seems to be co-expressed with the La-related protein7 (LARP7) host gene in several different mouse tissues examined [27, 28]. The previous studies show that miR-302 inhibits the proliferation of endometrial cells with inhibiting CDK1 and Cyclin D1 expression in MCF7 and HepG2 [42, 43]. Our current studies indicated that p100 could increase LARP7 transcription, which hosts miR-302d in the intron, and p100-mediated activation of miR-302d expression was crucial for its suppression of Cyclin D1 protein translation in bladder cancer cells. We also found that activation of LARP7/miR-302d was mediated by the increased phosphorylation of transcription factor CREB at Ser133, which was due to suppression of PHLPP2 expression by p100.

PHLPP2 is a well-characterized phosphatase for de-phosphorylation of Akt [44]. Our previous studies show that B[a]P/B[a]PDE exposure results in PHLPP2 suppression in both *in vitro* human lung epithelial cells and in *in vivo* mouse lung tissues, which consequently leads to c-Jun activation, TNFα induction, lung inflammation and carcinogenesis [45]. The results observed from current studies by either lost-function (knockout or knockdown) or gain-function (overexpression) clearly indicated that p100 expression was able to inhibit PHLPP2 expression, which increased CREB phosphorylation at Ser133 and consequently leading to miR-302d induction and Cyclin D1 protein translation inhibition. Although the mechanism underlying p100 inhibition of PHLPP2 expression is still under investigation, p100 acting as a tumor suppressor by initiating a novel PHLPP2/CREB/miR-302d/Cyclin D1 axis in regulation of anchorage-independent growth as illustration in Figure 8, together with our new discovery of p100 suppression in bladder cancers provides significant insights into the understanding of p100 in the processes of pathology in human bladder carcinogenesis. A complete understanding of p100 function in the pathogenesis of cancer development will spur the development of efficacious preventive and therapeutic approaches for controlling p100-suppressed cancers.

## MATERIALS AND METHODS

### Reagents, cell lines and transfection

Chemicals of MG132 and protein synthesis inhibitor cyclohexamide (CHX) were purchased from Calbiochem (San Diego, CA, USA). The dual luciferase assay kit was purchased from Promega (Madison, WI, USA). TRIzol reagent and SuperScript^™^ First-Strand Synthesis system were bought from Invitrogen (Grand Island, NY, USA). PolyJet^™^ DNA *In Vitro* Transfection Reagent was purchased from SignaGen Laboratories (Rockville, MD, USA). Both miRNeasy Mini Kit and the miScript PCR system for miRNA detection were bought from QIAGEN (Valencia, CA, USA). The antibodies against Cyclin D1, Cyclin E, Cyclin A, E2F1 and β-Actin were purchased from Santa Cruz Biotechnology (Santa Cruz, CA, USA). The antibodies against glyceraldehyde 3-phosphate dehydrogenase (GAPDH), S6 ribosomal protein, phospho-S6 ribosomal protein at Ser235/236, phospho-S6 ribosomal protein at Ser240/244, CREB, phospho-CREB at Ser133, c-Jun, phospho-c-Jun at Ser73, phospho-p38 MAPK at Thr180/Tyr182, phospho-MKK3/MKK6, phospho-SEK1/MKK4, SEK1/MKK4, PP2A-A, PP2A-B were bought from Cell Signaling Technology (Beverly, MA, USA). The antibodies against LARP7 and PHLPP2 were purchased from Bethyl Laboratories (Montgomery, TX, USA). The anti-p100 antibody used in immunohistochemistry staining (IHC) was purchased from Abcam (Catalog NO.ab131539, Cambridge, MA) and specifically recognizes p100, while antibody against p100 used in Western Blots was bought from Cell Signaling Technology (Catalog NO.4882, Beverly, MA, USA) and recognizes both p100 and p52. Primary p100+/+ and p100−/− mouse embryonic fibroblasts (MEFs) were immortalized according to the modified 3T3-immortalized protocol [46]. Human bladder cancer cell line UMUC3 and T24 cells were described in our previous studies [47, 48]. All cell lines were authenticated 4 to 6 months before use by using a short tandem repeat method. In all of the experiments conducted in this study, cells were synchronized by incubation in 0.1% fetal bovine serum (FBS; Nova-Tech, Grand Island, NE) containing medium overnight and cultured in 10% FBS medium for another 3-6 h before collection for further analysis unless specifically noted.

The shRNA constructs specific targeting mouse and human p100 and mouse CREB were purchased from Open Biosystems (Thermo Fisher Scientific, Pittsburgh, PA). Plasmids pBabe-puro p100 (1-900)/p52 (1–442) were generously provided by Dr. Han-Fei Ding (Cancer Center, Georgia Regents University, Augusta, GA) [19] and has been used in our published studies [49]. Mouse cyclin d1 3′-UTR luciferase reporter and cyclin d1 3′-UTR point mutation luciferase reporter (miR-302d binding site was mutated) were cloned into the pMIR report luciferase vector. Construct expressing miR-302/367 was obtained from Addgene (Cambridge, MA). Anti-miR-302d and its control vector were purchased from Genecopoeia (Rockville, MD). Mouse LARP7 promoter luciferase reporter and its mutations of CREB binding site were cloned into the PGL3 basic luciferase vector. The primers used in plasmid construction were listed in the Table S1. All new plasmids were verified by DNA sequencing. All of transfections were performed using PolyJet^™^ DNA *in vitro* transfection reagent (SignaGen Laboratories, Rockville, MD) according to manufacturer's instructions. Stable transfectants were selected with puromycin, hygromycin B or G418 for 3~4 weeks, and then cultured in regular medium for at least two passages prior to utilization for further experiments.

### Human bladder cancer tissue samples

The human bladder cancer tissues were collected between January 2013 and December 2013 from Wuhan Union Hospital, Tongji Medical College with informed consent and Ethics Committee's approval by the Medical Ethics Committee of Wuhan Union Hospital, Tongji Medical College of Huazhong University of Science and Technology (Wuhan, P.R. China). Paired human bladder carcinoma tissues and their adjacent normal bladder cancer tissues were acquired from patients receiving radical cystectomy. Adjacent normal bladder tissues were obtained from regions outside the tumor margin (> 5 cm). All tissues were macro-dissected within 15 minutes after surgical resection, and confirmed by pathological analysis of sequential frozen section, and then stored at −80°C until use. The stage and grade of the tumors were evaluated in line with the Union for International Cancer Control 2010 TNM system [50] and 2004 WHO classification system, respectively.

### *N*-butyl-*N*-(4-hydroxybutyl) nitrosamine–induced mouse bladder cancer model and immunohistochemistry paraffin (IHC-P)

All animal procedures were approved by the Committee on Animal Resources of the New York University School of Medicine and in according with NIH guidelines. The C57BL/6 male mice (*n* = 10/group) at age of 3–4 weeks were supplied ad libitum with tap water containing 0.05% BBN (TCI America, Portland, OR) in opaque bottles for 23 weeks, while negative control mice received regular tap water. The drinking water was prepared freshly twice a week, and consumption was recorded to estimate BBN intake. Mice were sacrificed at age week 23 of the experiments, and bladders were harvested and preserved in paraffin for pathological analysis and immunohistochemistry staining (IHC).

Bladder tissues obtained from the sacrificed mice specimens were formalin-fixed and paraffin-embedded. Immunohistochemistry staining (IHC) was performed to evaluate p100 expression between in both BBN-induced invasive bladder cancer tissues and negative control bladder tissues using antibodies specific against p100 (Abcam, Cambridge, MA) together with IHC kit based on the protocol instruction as described in our studies previously [45]. The resultant immunostaining images were captured by using the AxioVision Rel.4.6 computerized image analysis system (Carl Zeiss, Oberkochen, Germany). p100 protein expression levels were analyzed by calculating the integrated optical density per stained area (IOD/area) using Image-Pro Plus version 6.0 (Media Cybernetics, MD, USA).

### Reverse transcription-polymerase chain reaction (RT-PCR) and quantitative PCR assay

RT-PCR was performed to examine the expression level of Cyclin D1 and LARP7 mRNA. Total RNA was extracted from the cells using Trizol reagent (Invitrogen, Carlsbad, CA). Total cDNAs were synthesized by ThermoScript TM RT-PCR system (Invitrogen, Grand Island, NY). The mRNA amount presented in the cells was measured by semi-quantitative RT-PCR. The results were imaged with Α Innotech SP image system. Quantitative RT-PCR assay was performed to examine the expression level of mature miRNAs. The primers used in this study were listed in Tables 1 and 2.

**Table 1:** The details of primers used in this study

Mouse Cyclin D1	Forward: 5′-GTG CCA TCC ATG GGG AA-3′Reverse: 5′-GGA TGG TCT GCT TGT TCT CA-3′
Mouse LARP7	Forward: 5′-TAT GGA AGA AAG CAC TAA GAG A-3′ Reverse: 5′-CAC ATA TAC CGT GCG TTC CTC C-3′
Mouse β-Actin	Forward: 5′-ATA TCG CTG CGC TGG TCG TC-3′Reverse: 5′-AGG ATG GCG TGA GGG AGA GC-3′
Mouse GAPDH	Forward: 5′-ATC AAG AAG GTG GTG AAG CAG GCA-3′Reverse: 5′-TCT CTT GCT CAG TGT CCT TGC TGG G-3′
Mouse Cyclin D1 3′UTR	Forward: 5′-CCG CTC GAG AAA TGT ACT CTG CTT TGC TGA-3′Reverse: 5′-CTA GAC TAG TGA TCG CCA TCA GGG TCC CAG GA-3′
Mouse Cyclin D1 3′UTR miR-302d Point Mutation Primers	Sense: 5′-AAA CCA TTC CAT TTC AAT CGT CTT TTG GTC AGC TAG CT-3′Antisense: 5′-AGC TAG CTG ACC AAA AGA CGA TTG AAA TGG AAT GGT TT-3′
Mouse LARP7 promoter	Forward: 5′-CCG CTC GAG CAA TCA GTT TTT AGT CAG CAT-3′ Reverse: 5′-CCC AAG CTT GGG AGC CAC GCG GTG ACT GC-3′
Mouse LARP7 promoter CREB Point Mutation Primers	Sense: 5′-CCA ACT TAA GTG ACG TCT ACA AGC AGG GAC GGT ACC-3′Antisense: 5′-GGT ACC GTC CCT GCT TGT AGA CGT CAC TTA AGT TGG-3′

**Table 2:**
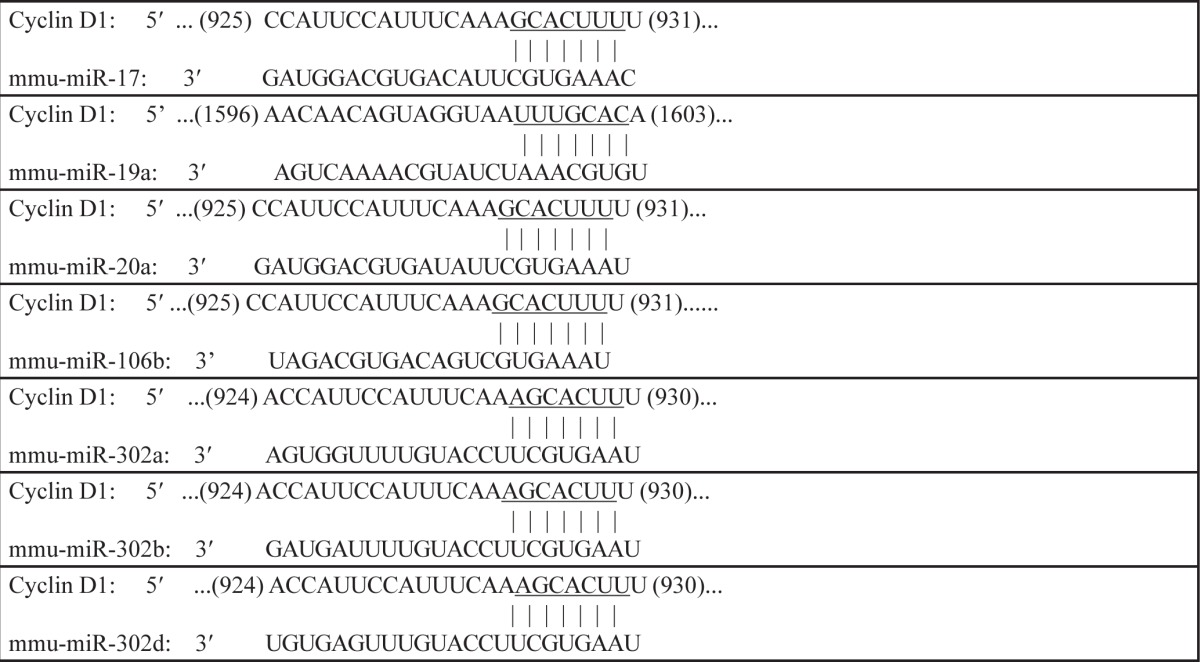
Sequence alignments of cyclin D1 3′-UTR with seed regions of putative microRNAs

### Flow cytometry

Cells were seeded into 6-well plates and cultured until they were 70–80% confluence. The cell culture medium was replaced with DMEM containing 0.1% FBS and cultured for 12 h, and the cells were cultured in DMEM with 10% FBS for 3 h. The cells were washed with ice-cold phosphate-buffered saline (PBS) 2 times, and then fixed in ice-cold 70% ethanol at −20°C overnight. The cells were washed with PBS 3 times, and cell apoptosis was analyzed using flow cytometry (Beckman, Indianapolis, USA) after staining for 15 min with propidium iodide (PI) buffer (0.1% Triton ×-100, 0.2 mg/ml RNase A, 0.05 mg/ml propidium iodide). DNA content was determined by flow cytometry using Epics XL flow cytometer (Beckman Coulter Inc, Miami, FL) and EXPO32 software.

### Anchorage-independent growth assays

Soft agar colony formation assay was performed as described previously [51]. Briefly, 3 ml of 0.5% agar in basal modified Eagle's medium (BMEM) supplemented with 10% FBS was layered onto each well of 6-well tissue culture plates. UMUC3 cells or T24 cells (1 × 10^4^ cells) were suspended in 1 ml of normal medium were mixed with 2 ml of 0.5% agar in basal modified Eagle's medium supplemented with 10% FBS, and 1 ml of mixture was added into each well on top of the 0.5% agar layer. Plates were incubated at 37°C in 5% CO_2_ for 2–3 weeks, and the colonies were scored and presented as number of colonies per 10^4^ cells.

### [^35^S] Methionine pulse protein synthesis assays

Cells were incubated with methionine-cysteine-free DMEM (Gibco-BRL, Grand Island, NY, USA) containing 2% dialyzed fetal calf serum (Gibco-BRL) and 10 μM MG132 for 30 minutes, and then incubated with 2% FBS methionine-cysteine-free DMEM containing ^35^S labeled methionine/cysteine (250 μCi per dish, Biomedicals, Inc., Irvine, CA) for the indicated time periods. The cells were extracted with lysis buffer (Cell Signaling) containing complete protein inhibitor mixture (Roche) on ice and 500 mg total lysate was incubated with anti-Cyclin D1 antibody-conjugated agarose beads (R&D Systems, Minneapolis, MN, USA) overnight at 4°C. The immunoprecipitates were washed with the cell lysis buffer five times, heated at 100°C for 5 min and subjected to sodium dodecyl sulfate polyacrylamide gel electrophoresis. The membranes were then subjected to autoradiography for determination of the newly synthesized ^35^S-labeled Cyclin D1 protein as described in our studies previously [52].

### Western blotting

Cells were seeded into 6-well plates and cultured until 70%–80% confluence. The cells were then extracted with cell lysis buffer (10 mM Tris-HCl, pH 7.4, 1% sodium dodecyl sulfate, 1 mM Na_3_VO_4_ and proteasome inhibitor). The protein concentration was determined using Nano Drop 2000 (Thermo Scientific, Holtsville, NY, USA). The cell extract (60–80 μg/sample) was subjected to Western Blotting with specific antibodies as described in our previous studies [49]. The densitometry analyses of the protein bands were performed using the ImageQuant 5.2 software (GE Healthcare, Pittsburgh, PA).

### Luciferase assay

MEF and UMUC3 cells were transfected with the indicated luciferase reporter construct in combination with the pRL-TK vector (Promega, Madison, WI). The transfectants were seeded into 96-well plates and cultured for 12 hours. The cells were then extracted with luciferase assay lysis buffer (Promega, Madison, WI) and then subjected to determination of luciferase activity using the luciferase assay system (Promega Corp., Madison, WI) with the microplate luminometer LB 96V (Berthold GmbH & Co. KG, Bad Wildbad, Germany). The luciferase activity was normalized to internal control TK activity based on the manufacturer's instructions.

### Statistical analysis

The student's *t-test* was used to determine significant differences and *p* < 0.05 was considered as a significant difference between compared groups.
